# “Post-Protean” Public Health and the Geroscience Hypothesis

**DOI:** 10.14336/AD.2023.0721

**Published:** 2024-04-01

**Authors:** Colin Farrelly

**Affiliations:** Department of Political Studies, Queen’s University, Kingston, Ontario, K7L 3N6, Canada.

**Keywords:** aging, disease, Geroscience hypothesis, healthspan, public health

## Abstract

Despite unprecedented investments in public health and biomedical research, improvements in life expectancy and healthy life expectancy have stagnated in the United States. Part of the reason for this development can be traced back to the influence of “Protean” over “Post-Protean” public health, the names that can be given to two contrasting visions of public health advanced in the early twentieth century. Protean public health prescribes “waging a war” against disease and was successful in reducing the early-life mortality risks from infectious disease. But Protean public health has proven less effective in improving the quality of life of older persons. Post-Protean public health prioritizes the *experimental method* and research into the indirect methods of improving health. It articulated a vision of public heath that was given a more concrete specification by Alex Comfort in what is now referred to as the *Geroscience Hypothesis*. To improve the health prospects of aging populations the dominance of Protean public health must be relaxed, to enable the benefits of Post-Protean public health to be realized. Doing so means shifting public health’s aspirations towards increasing the *healthspan* vs “saving lives” by extending the duration of time older persons can survive by managing the multi-morbidities of late life.

Between 1959 and 2016, US life expectancy increased from 69.9 years to 78.9 years [1]. However, further increases in US life expectancy have not only stalled, life expectancy has also actually declined over the past few years. And a much more important measure- healthy life expectancy at birth- has only increased by 0.3 years (65.8 to 66.1 years) (www.who.int/data/gho/data/indicators/indicator-details/GHO/gho-ghe-hale-healthy-life-expectancy-at-birth) for the US population from 2000 to 2019. Never before in human history has so much funding been invested in public health and biomedical research- the NIH invests billions each year in pathology research and is the largest public funder in the world-while a country’s population also experiences a reduction in life expectancy and only marginal improvements in healthy life expectancy.

The predicament of contemporary US life expectancy and healthy life expectancy can, at least in part, be traced back to two different visions of public health that were initially put forth over a century ago. Had both visions of public health been aggressively pursued in the twentieth century the current state of life expectancy and healthy life expectancy in the US may have been more promising than it currently is. However, because of the myopic fixation on preventing disease and prioritizing pathology research, only one vision of public health was aggressively pursued throughout the twentieth century. A consequence of this was that geroscience was marginalized as a potential tool for public health. This Perspective piece explains some of the intellectual history behind the two visions of public health articulated by public health pioneers in the early twentieth century, and how the dominance of one of those visions created inertia that constrained realizing the benefits of the *Geroscience Hypothesis*- the conjecture that strategies designed to modify the biological drivers of aging will not only slow the progression of biological aging but will also prevent or delay the onset of multiple chronic diseases [2].

**Figure 1.**
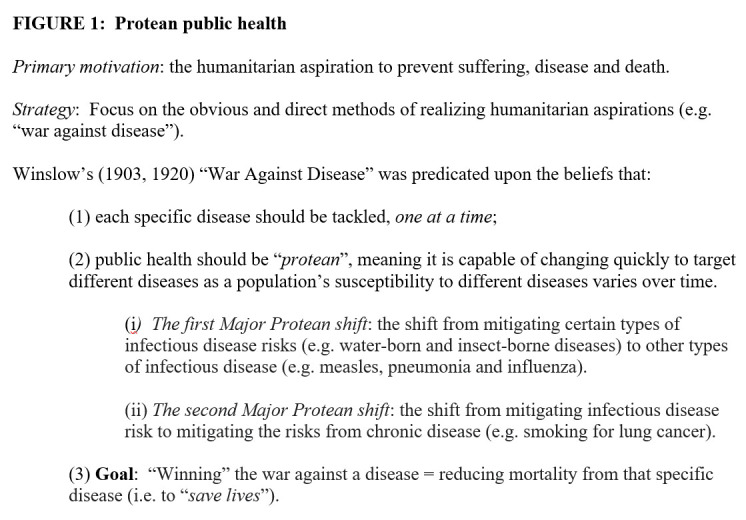
Protean public health.

## Two Visions of Public Health

**Figure 2.**
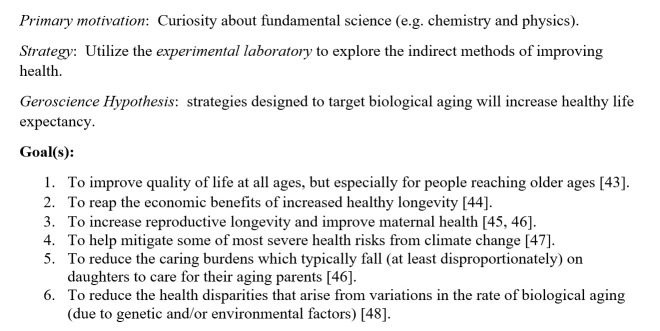
Post-Protean Public Health.

The two visions of public health can be called “Protean” (Fig. 1) and “Post-Protean” (Fig. 2) public health. Public health is the science and art of preventing disease and prolonging life [3]. “Protean” public health is the scientific approach first developed to combat the infectious diseases responsible for high rates of early-life mortality. As such it is motivated by humanitarian aspirations (e.g., to save lives) and focuses on the obvious and direct methods of preventing and treating disease. Protean public health inspires a “war against disease” mentality, a mindset that:

1. proposes tackling each specific disease, *one at a time*;
2. is “*protean*”, meaning it is capable of changing quickly, so it will shift its aim to mitigating different diseases as a population’s susceptibility (increases or decreases) to different diseases varies over time; and
3. equates “winning” the war against a disease with reductions in mortality from that disease (i.e., to “save lives”).

The most prominent advocate of Protean public health was the public health pioneer C-E.A Winslow (1877-1957), and the three tenets of Protean public health identified above are expressed in his 1903 essay “The War Against Disease” (www.theatlantic.com/magazine/archive/1903/01/the-war-against-disease/638202/) and his influential 1920 *Science* article entitled “The Untilled Fields of Public Health” [3].

A second vision for public health and medicine was offered by Christian Herter (1865- 1910) in his 1910 JAMA Address entitled “Imagination and Idealism in the Medical Sciences” [4,5]. Herter maintained that the humanitarian aims of medicine- the idealism that delights in the relief of human suffering and disability- was, while integral to medicine, a source of weakness as well as a strength. It was a source of weakness for it would lure the medical profession into the myopic “war against disease” mentality that Protean public health advocates. Herter remarked:

For he who would answer the calls of the sick must resort to direct methods and must generally tread the paths of the obvious. He has not time to turn aside to the indirect ways of winning the citadel, nor, indeed, is he likely to be in that frame of mind which urges to such an approach; he is preoccupied with the crying needs of the suffering or dying man committed to his charge. Yet it is growing every day clearer that the progress of the medical sciences depends in a remarkable degree on discoveries made by indirect methods—that is, by methods not looking to the immediate relief of disease [4].

Post-Protean public health thus emphasizes the importance of also attending to the *indirect methods* of promoting health (vs the direct methods of preventing specific diseases), the experimental laboratory research that transcends the limitations of the pathology-focused paradigm of medical science. Herter believed the experimental laboratory was a vital public health tool that was neglected by medical science in the early twentieth century. His Post-Protean vision of public health was given a more concrete expression in 1969 by the gerontologist Alex Comfort (1969) [6], who was the first to articulate the Geroscience Hypothesis.

## Winslow’s Protean Public Health and The War Against Disease(s)

The early twentieth century public health pioneer C-E.A Winslow, who became the first Chairman of Yale University’s School of Public Health when it was founded in 1915, championed three central ideas which would become among the foundational intellectual premises of public health and biomedical research for over a century. The first idea was articulated in his 1903 essay entitled “The War Against Disease”. There Winslow maintained that science must commit itself to a “war against disease”, and that war must be waged against each specific disease, *one at a time*. He remarked:

Each disease must be fought after its own kind. For smallpox, vaccination; for diphtheria, antitoxin inoculation; for typhoid fever, the protection of food supplies; for yellow fever, the destruction of mosquitoes; for tuberculosis, the disinfection of sputum; for cholera infantum, the cooking of milk. (https://www.theatlantic.com/magazine/archive/1903/01/the-war-against-disease/638202/).

Consider, for example, the war against the infectious disease cholera. In the nineteenth century the US had three major cholera outbreaks- in 1832, 1849 and 1866 [7]. The very first article published [8] in the *Journal of the American Public Health Association* (now *American Journal of Public Health*) in 1911 focused on the public health strategies of preventing Asiatic cholera. These included detaining and quarantining for 5 days all emigrants bound for the United States from regions of the world deemed at risk of Asiatic cholera. The food and water supplies of their ships were also investigated. Safe water, hygiene and sanitation helped reduce the health threats from water-borne diseases like cholera. In their study on the effect of sanitation and clean drinking water on child mortality and life expectancy in 100 countries, Ummalla et al. [9] conclude that access to improved drinking water sources and improved sanitation facilities significantly reduces the child mortality rate and increases life expectancy.

The second, related, idea Winslow championed, this time in his influential 1920 *Science* article “The Untilled Fields of Public Health”, was that the public health movement had to be *protean* (i.e., capable of change). However, Winslow conceived of public health’s ability to change as one that functioned only *within* the parameters of the “war against disease” paradigm [10]. So, while the specific targets (e.g., water-borne infectious diseases, vector-borne infectious diseases, chronic diseases) of public health may change over time, as different diseases become more or less prevalent within a population, the primary focus of public health and medicine was to remain focused on the goal of disease control.

As the aims of sanitation are approximately realized in a given community, the attention of the health official turns from the water-borne and insect-borne diseases to the more subtle and more baffling maladies that are spread by direct contact from one individual to another. As typhoid, cholera, plague and typhus fever approach the vanishing point, measles, pneumonia and influenza become relatively more and more important. (https://www.theatlantic.com/magazine/archive/1903/01/the-war-against-disease/638202/)

The first Major Protean shift, that Winslow notes in the passage above, was the cognitive flexibility necessary to shift public health priorities and resources from mitigating certain types of infectious disease risks (e.g., water-born and insect-borne diseases) to protecting against other types of infectious diseases (e.g., measles, pneumonia and influenza). In 1946 the Communicable Disease Center (CDC) was first created, and its primary mission was to prevent the spread of malaria by waging a war on mosquitos.

The second Major Protean shift was originally articulated by Winslow’s mentor Hermann Biggs (1859-1923). Biggs was the general medical officer of the Department of Public Health for New York City, and famously coined the slogan “public health is purchasable”. This slogan expresses a core conviction of Protean public health as it maintains that disease was mostly an avoidable state-of-affairs; something that only exists and persists because of unfavourable economic and living conditions. This belief was an expression of Protean idealism (and “folkbiology” [11]) vs an empirically justified conjecture predicated upon intimate knowledge of human biology (e.g., disease genetics, or evolutionary biology). Anticipating that success would be realized with mitigating the health risks posed by infectious diseases that caused high rates of early-life mortality, Biggs remarked:

The future development of public health work will include the opening up of a field in which little or nothing has been done. Systematic attack will be made, principally, by education of the public in prophylactic measures, against those diseases in middle and later life which are not infectious, and which have hitherto been regarded as entirely outside the sphere of public intervention [12].

Protean public health’s most significant successes came from preventing infectious diseases (e.g., cholera, TB, yellow fever, malaria, etc.) and, on January 11, 1964, nearly half a century after Biggs recommended applying Protean public health to non-infectious diseases, the American Surgeon General Luther Terry released the first report of the Surgeon General’s Advisory Committee on Smoking and Health. Smoking was identified as a cause of lung cancer. And with the National Cancer Act of 1971 and President Richard Nixon’s declaration of a “war on cancer”, the war against disease officially expanded its scope to aspiring to eliminate chronic disease. The CDC officially changed its name to its current name- Centers for Disease Control and Prevention- in 1992.

The health prospects of aging populations were thus placed in the hands of the humanitarian idealism that shaped Protean public health. And while improvements were made in delaying the age of death from chronic disease, no disease has been cured or eliminated. Protean public health has not proved as successful in preventing or eliminating chronic disease as it has for infectious disease. But this fact has only emboldened Protean public health and medicine, as significant amounts of funding continue to be invested each year in pathology research and the passing of measures like the National Alzheimer’s Project Act (2011) and the 21st Century Cures Act (2016) offer the renewed promise that victory in the war against disease is imminent.

**Figure 3.**
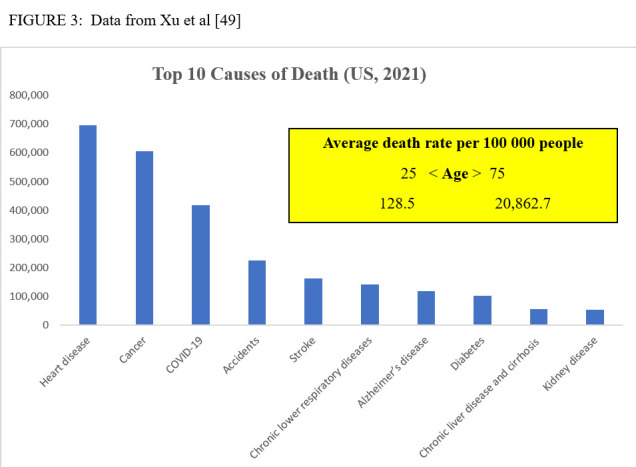
Data from Xu et al [49].

Even if a cure for one or more of the diseases of late life could be achieved, the health dividends would be marginal because, as Olshansky (2016) notes, it can mean more debilitating diseases can become more prevalent because that hazard in old age is not that one disease simply replaces another, but that the new diseases can often be more debilitating [13]. Figure 3 reveals the steep uphill battle the “war against disease” faces for today’s aging populations. The belief that the most prevalent diseases of late life could be eliminated reflects the humanitarian idealism Herter identified back in 1910. But public health’s preoccupation with “the crying needs of the suffering or dying man” led to the marginalization of the study of the indirect methods of improving health. The latter is the primary concern of “Post-Protean” public health.

## Post-Protean Public Health: Age Retardation

A second vision for public health and medicine, one that transcended the myopic fixation on pathology research, was initially articulated by Christian Herter in 1910. Herter received his M.D. from Colombia University at age 19 and later became Professor of the Department of Pharmacology and Therapeutics at Colombia University. He also co-founded the *Journal of Biological Chemistry* in 1905. Tragically Herter developed a wasting nervous illness (possibly myasthenia gravis [14]) and died at age 45, a few months after the publication of his 1910 JAMA Address.

Herter emphasized the significant relation between laboratory science and medicine, something unexplored and under-appreciated during his lifetime. He actually had a laboratory designed for a large part of the upper level of his house when it was built in 1893 (https://www.nytimes.com/1910/12/08/archives/physicians-mourn-dr-herters-death-distinguished-scientist-who.html). In contrast to the immediate urgency and pathology-focus of the call to a “war against disease”, Herter’s vision of scientific innovation was one which celebrated instead curiosity and placed a strong emphasis on the fundamental sciences (like physics and chemistry). Herter believed the fundamental sciences “could come to the aid of physiology, biology, pathology and psychology”. He described his vision of medicine in the early twentieth century as follows:

I like to think of medicine in our day as an ever broadening and deepening river, fed by the limpid streams of pure science. The river at its borders has its eddies and currents, expressive of certain doubts and errors that fringe all progress; but it makes continuous advances on the way to the ocean of its destiny. Very gradual has been the progress of its widening and deepening, for it is a product of human ingenuity and artifice, and only skilled engineers could direct the isolated currents of science into the somewhat sluggish stream of medical utility [4].

Geroscience is arguably the exemplar example of scientific progress that has been gradual and expressive of doubts and errors, the product of ingenuity and artifice over nearly a century of scientific research, and only something skilled engineers could direct to the sluggish stream of medical utility. Sadly, Herter died in 1910, so he did not live to see the significant scientific insights yielded by laboratory studies on the biology of aging over the course of the twentieth century. The idealism and imagination behind the Geroscience Hypothesis coheres with Herter’s Post-Protean paradigm of public health. A paradigm which encouraged laboratory science and a focus on indirect methods of improving health vs the obvious and direct methods of the “war against disease”.

Experiments on short-lived species like rodents, mice and fruit flies were the laboratory animals of choice for the initial research on the biology of aging. In the 1930s experiments in caloric restriction in rodents demonstrated that aging was malleable, and that caloric restriction could increase lifespan. Such research was not pathology research. Unlike the search for a cure for malaria or tuberculosis, restricting the food intake of mice and rats fell outside the scope of concern of Protean public health. And it was not research that would yield an immediate practical application to clinical medicine. It would take the path of the flow of the deepening river Herter described above, with its eddies, currents, doubts, and errors, and yet making continuous advances towards its destiny.

In the 1980s the “era of genetic manipulations in aging” [15] in invertebrates and mice demonstrated that genetic mutations lead to increased life span. And now geroscience has entered the exciting stage of pharmacological and pharmaceutical interventions in aging. For nearly a century geroscience has been the unsung exemplar example of Post-Protean public health. It prioritized laboratory science and the examination of indirect ways of promoting health vs attending to the immediate relief of disease (the sole focus of Protean public health). Many of the qualities of mind Herter attributes to “the experimentalists” apply to those who study the biology of aging. Accounts of the evolutionary explanation of aging [16], for example, are illustrative of Herter’s description of researchers concerned more with “function rather than structure”. And scientists that test, through experimentation, the hypothesis that aging is malleable have “minds, far from being dismayed by the speculative aspects of their studies, invite such speculation so long as it is severely controlled by frequent appeals to facts won by experiment”. The countless number of experiments researchers have done on trying to manipulate aging and lifespan in species as varied as rodents, worms, and fruit flies- through different types of interventions (e.g. calorie restriction, genetic manipulation, etc.)- are indicative of the type of person Herter had in mind when he remarked: “…their thoughts in leisure hours, as in the hours of work, turn always restlessly and uncontrollably in the same direction—to the planning of new experiments designed to answer the questions uppermost in consciousness, questions having nearly always to do with the phenomena of living beings”.

The Geroscience Hypothesis was first championed by the gerontologist Alex Comfort (1920-2000) in his 1969 article entitled “Longer Life by 1990?” [6] and many many others have also advanced a similar line of argument [17, 18, 19, 20, 21, 22, 23]. Comfort predicted the predicament the US (and other developed countries) now face in terms of the stifling of the health and longevity returns from the current (Protean) approach to public health. Comfort conjectured that there was a “biological wall” that would limit the gains likely to be realized by further improvements in medicine and living conditions. What was needed, he argued, was a “systems breakthrough” approach to health promotion which was very distinct from the approach which championed sociomedical advances. Only a Post-Protean idealist, with an engineer’s mindset, could propose a “systems breakthrough” approach to longevity.

Comfort boldly predicted that, even if the 1969 level of US government investment in R & D were maintained, the first experiment on delaying aging in humans was certain to have taken place by 1975 and it was likely that the discovery of some agent that reduced aging in humans would be known by 1985. And finally, he believed the lifespan increase of such an agent could be as much as 20%, possibly more. Unfortunately, Comfort’s prediction concerning the speed at which the Geroscience Hypothesis would be realized did not come to fruition. But Comfort’s plea for shifting science’s focus away from pathology towards targeting the aging process itself echoed Herter’s arguments and the Post-Protean vision of public health.

What Comfort perhaps failed to appreciate was how strong the grip of what Herter identified as the humanitarian impulse of the medical sciences would be. The latter maintained that the sole function of public health and medicine was to “save lives”, not improve health. To “answer the calls of the sick” one must pursue the “direct methods and must generally tread the paths of the obvious”. As the US population aged, and, because in 1951 all state and federal agencies in the United States were required to adopt a standard list of contributing and underlying causes of death (eliminating “old age” as a cause of death) [24] there was little room for imagining any public health success that could not be equated with simply reducing the mortality risks of specific diseases. The humanitarian idealism of Protean public health would remain fixated on pathology research because, with no one officially suffering or dying from aging, research on the biology of aging was not an obvious route to pursue to benefit the vulnerable. With the greatest risk factor for the leading cause of death ignored [25], the dominance of Protean public health in the second half of the twentieth century was solidified. The only way this dominance could be dislodged was if the following three things occurred *concurrently*:

1. stifled increases in life expectancy;
2. the failure to cure the most prevalent diseases of aging that are responsible for most late-life mortality; and
3. significant advances in geroscience that demonstrated that it could have real clinical applications.

All three of these developments are true today, which makes the commitment to only prioritize Protean public health untenable. To continue the sole path of Protean public health is to commit to the goal of simply extending the period of time in late life that people can survive managing multi-morbidity, frailty and disability. The compassionate goal for aging populations is to instead invest a proportionate number of resources and energies into the goal of increasing the *healthspan*, to improve quality of life at all ages (especially for older persons). If Protean idealism ignores the biology of aging it will continue its myopic pursuit of trying to increase lifespan further by targeting each specific disease of late life at exorbitant costs for marginal benefits to healthy life expectancy.

## Concerns and Challenges

It is important to acknowledge that there are legitimate grounds for some scepticism concerning the magnitude of the health benefits the Geroscience Hypothesis is likely to yield, as well as concerns about the fair diffusion of an aging innovation and the prospects of transcending the inertia of the pathology-focused approach to medicine. For example, some aging scientists caution against over-enthusiasm or scientism and the confidence that the results in animal models can be easily translated in humans [26]. Caloric restriction, for example, is a feasible intervention to impose upon laboratory animals as food intake can be controlled, but CR is not something humans could be expected to comply with, at least for prolonged periods of time. Furthermore, the magnitude of the impact CR-mimics has on the healthspan of animal models might differ from the magnitude they could have in humans.

One promising approach, that aspires to redress the *information gap* in translating findings from interventions in animal models to humans, is the Dog Aging Project (https://dogagingproject.org/), a long-term longitudinal study of aging in tens of thousands of dogs. Companion dogs (unlike laboratory animals) and humans share physical and chemical environments, as well as have similar experiences in terms of functional decline and disease with aging, thus the results from studying the aging of dogs could more readily translate to findings for humans [27].

Some bioethics [28,29] have expressed the concern that an aging intervention could exacerbate existing health inequalities if it were only accessible to the affluent, such as persons living in developed countries. Of course, this problem arises with any health innovation, as new drugs or medical procedures will (at least for some period of time) be available only to those who can afford them. Over time the costs of such interventions typically decline, and they become more accessible. For example, when the patent on a drug expires and low-priced generic versions can be manufactured. The speed at which an aging innovation could be widely diffused for the global population will really depend on the details of the kind of intervention it is. A promising Post-Protean public health strategy, in terms of both safety and the prospects for the fair diffusion of an aging intervention, is the re-purposing of FDA drugs that are both off-patent and have an extensive track-record for safety in treating a disease [30]. For example, drugs like metformin and rapamycin. The former has been utilized as a pharmacological intervention to control type 2 diabetes for decades and has also been shown to improve both healthspan and lifespan in different animal models [31]. And rapamycin is a drug used to help prevent the rejection of transplanted organs for patients undergoing organ transplant and there is strong evidence for rapamycin’s effect on aging and age-related diseases in mice [32].

Is physical exercise an example of post-Protean public health, given that it can increase healthspan and helps with the prevention of a multiple of pathologies? I believe the answer is “yes”. While there is a consensus on the issue that exercise can increase healthspan, there is disagreement on whether it alters the mechanisms of aging. Austad contends that, while exercise has been shown to increase mean longevity in both rats and people, it is not generally considered an intervention that slows aging because it does not increase maximal survival (though he notes many researchers are now re-thinking the over-reliance on longevity as the canonical metric of aging) [2]. Caloric restriction, for example, is able to delay aging processes that increase both mean and maximum lifespan whereas exercise primarily increases healthspan [33]. Some contend that exercise is a potent anti-aging and anti-chronic disease medicine and should be examined further as a potential senolytic medicine (which targets senescent cells) for aging and various diseases [34]. However, one thing that is now clear, many decades after the known preventative health benefits of exercise [35], is that poor compliance (especially in the elderly population) makes this challenging to apply [22]. Like trying to combat the risks from infectious diseases by relying solely on behavioural interventions like hand washing, wearing face masks and isolating the infected (vs having the benefits of vaccines), to substantively improve the quality of life of older persons medical innovation that develops pharmacological interventions to slow aging will be necessary, in addition to encouraging more exercise across the lifespan.

A major hurdle Post-Protean public health faces is how to facilitate the regulatory shift needed to move away from the model of drug development designed to target only specific diseases, to a regulatory framework that is inclusive of drug innovation designed to target aging itself. The so-called “Hallmarks of Aging” identify and categorize the cellular and molecular hallmarks of aging [36], which are: genomic instability, telomere attrition, epigenetic alterations, loss of proteostasis, deregulated nutrient sensing, mitochondrial dysfunction, cellular senescence, stem cell exhaustion, and altered intercellular communication. More recent additions to the hallmarks of aging include changes in composition of the microbiome, chronic inflammation, disabled macro-autophagy and stiffening of the extracellular matrix [37, 38, 39, 40]. These hallmarks help bring precision to the range of known biological mechanisms of aging that should be the focus of the development of safe and effective *gerotherapeuthics*- drugs that target pathways involved in aging with the aim of reducing the burden of aging-related diseases and increasing lifespan and healthspan [41].

One of the significant challenges of testing the geroscience hypothesis in humans is how to address concerns of safely when testing a gerotherapeutic in healthy volunteers. Experimental drugs designed to treat or manage a specific disease can point to the harms of non-intervention (e.g., disease progression) to provide a compelling justification for tolerating some potential risk of harm during the clinical trial phase of a novel drug. A critic might contend that any attempt to intervene in human aging, through experimental drug interventions, is inherently ethically dubious because aging itself is not a disease. Therefore, the critic might continue, a *precautionary* approach precludes ever testing gero-therapeuthics in healthy people because the potential harm/benefit calculation would not justify taking any risk.

But such a line of reasoning reveals how Protean public health suffers from an “aging status quo” bias. While aging itself may not be a disease, it is reasonable to assume aging poses plausible and serious threats to human health given it is the major risk factor for most diseases and disability. Resnick contends that, in medical decision-making, appeals to a precautionary principle- when uncertainty exists concerning the potential benefits and harms of a novel intervention- must not be overly risk-adverse [42]. He argues that a standard of “reasonableness” be invoked, one which prescribes that “one should take reasonable measures to prevent or mitigate threats that are plausible and serious.” Post-protean public health sees “normal aging” as a plausible and serious threat to people’s health, even if aging is not, strictly speaking, “a disease”.

The Longevity Biotechnology Association (LBA) is a non-profit organization created to foster innovation in medical research which targets the mechanisms of aging to promote healthspan (www.longevitybiotech.org/). LBA aspires to help foster collaboration among industry innovators and has proposed guidelines to help drug development transcend the limitations of protean medicine [37]. For example, LBA emphasizes the importance of ensuring that any potential aging interventions are backed by rigorous science vs non-rigorous evidence that simply exploits the public’s interest in increased healthspan. Gerotherapeuthics must meet rigorous standards for scientific evidence, for both safety and efficacy, and only then will the potential harm/benefit ratio shift in favour of intervening in aging versus maintaining the aging “status quo”.

## Conclusion

Protean public health provided significant improvements to the health and longevity of the US population in the twentieth century. It helped abate many of the infectious diseases responsible for early-life mortality. Diseases that once plagued the US population- like yellow fever, tuberculosis, diphtheria, smallpox, malaria, polio, etc.- have now been abated and replaced by the chronic diseases of late life.

Protean public health transferred the intellectual presuppositions of the “war against infectious disease” to the “war against chronic disease”. And while this strategy has also resulted in significant successes—such as delaying the onset of disease and age of mortality— it has also made our minds, in Herter’s words, “statical in conception”. Our minds can become so statical that we somehow convince ourselves that “success” in the war against disease is both achievable and desirable, despite the significant investments made each year in pathology research and the marginal returns to healthy life expectancy. The health and economic wellbeing of future generations will be significantly improved if public health commits itself to the aspiration to slow the rate of biological aging so the population can enjoy more healthy years of life. It is imperative that we shift from a Protean public health framework to a hybrid approach that gives greater priority to Post-Protean public health aspirations and innovations.
